# Modified ocular surface disease index as a screening criteria for dry eye syndrome presenting after successful dacryocystorhinostomy

**DOI:** 10.1371/journal.pone.0247168

**Published:** 2021-02-25

**Authors:** Tae Seen Kang, Jin Cho, Jaeyoung Kim, Jae Yun Sung, Ju Mi Kim, Kyoung Nam Kim, Sung Bok Lee

**Affiliations:** 1 Department of Ophthalmology, Chungnam National University School of Medicine, Daejeon, Republic of Korea; 2 Department of Ophthalmology, Gyeongsang National University Changwon Hospital, Changwon, Republic of Korea; 3 Department of Ophthalmology, Chungnam National University Sejong Hospital, Sejong, Republic of Korea; Xiamen University, CHINA

## Abstract

**Objective:**

To find preoperative screening criteria for dry eye syndrome (DES) that present after successful endoscopic dacryocystorhinostomy (EDCR).

**Methods:**

We retrospectively analyzed medical records of 110 patients who underwent EDCR for nasolacrimal duct obstruction. DES diagnostic criteria were defined as tear break-up time (TBUT) less than 10 seconds, and ocular surface disease index (OSDI) score greater than 13 points. After EDCR, patients were divided into DES group and control group according to the DES diagnostic criteria. Preoperative OSDI score alone or in combination of preoperative TBUT and OSDI score were used to find screening criteria, which could discriminate the two groups preoperatively with a high positive predictive value (PPV). Criteria A was set same as the diagnostic criteria of DES, and Criteria B and C were set to improve PPV by increasing specificity while maintaining similar sensitivity to Criteria A.

**Results:**

Thirty patients (27.3%) were diagnosed with DES after EDCR, while 80 patients (72.7%) were normal. In patients with DES, preoperative TBUT was not different (p = 0.851), but OSDI score was significantly higher (p<0.001). Criteria A showed a sensitivity of 73.3%, specificity of 55.0%, and PPV of 38.0%. Criteria B and C excluded preoperative TBUT, which had no difference between the two groups, and set screening criteria with preoperative OSDI score alone. Criteria B (preoperative OSDI score of 19.6 points or more) showed a sensitivity of 75.0%, specificity of 60.6%, PPV of 41.7% and AUC of 0.739 (p < 0.001). Criteria C was consisted of 5 out of 12 OSDI items that showed significant differences between the two groups; blurred vision, reading, working with a computer, low humidity, and air conditioning. Criteria C (preoperative 5-item OSDI score of 24.4 points or more) was a better predictability, with a sensitivity of 75.0%, specificity of 71.3%, PPV of 49.5%, and AUC of 0.804 (p < 0.001). The AUC of Criteria C was significantly higher than that of Criteria B (p = 0.0037).

**Conclusion:**

DES occurred after successful EDCR in 27.3% of patients, and an OSDI questionnaire helped to screen DES. The predictability could improve using the modified OSDI score which showed noticeable difference in five OSDI items before and after EDCR.

## Introduction

Nasolacrimal duct obstruction (NLDO) is a disease that causes chronic epiphora and ocular discomfort. Dry eye syndrome (DES) is a multifactorial ocular surface disease characterized by a loss of homeostasis of the tear film, and accompanied by ocular symptoms, in which tear film instability and hyperosmolarity, ocular surface inflammation and damage, and neurosensory abnormalities play etiological roles [1].

NLDO and DES have a high prevalence, and share demographic characteristics, such as increasing prevalence with age and a higher incidence in females [2, 3]. In addition, both diseases share various ocular symptoms such as decreased vision due to irregular refraction caused by changes in the tear film, sensitivity to light, foreign body sensation, and stringy mucus around eyes. Exacerbation factors are also similar including exposure to dry, cold and windy conditions which make epiphora and ocular irritation symptoms worse.

According to Tear Film and Ocular Surface Dry Eye Workshop II (TFOS DEWS II), DES is diagnosed by symptoms and signs. Usually, the symptoms are evaluated by Dry Eye Questionnaire-5 (DEQ-5) or Ocular Surface Disease Index (OSDI), and the signs are assessed by tear film break-up time (TBUT), osmolarity, and fluorescein dye staining. In NLDO patients, tear film thickness increases [4], tear inflammatory cytokines increase [5], and tear osmolarity decreases [6]. Additionally, evaluating ocular surface staining in patients with NLDO is difficult due to thick tear meniscus. Because of these reasons, DES diagnostic tests have limitations and are less valuable in diagnosing DES when NLDO is combined. Because DES symptoms masked by NLDO symptoms can appear after surgery, patients who have not been diagnosed with DES before surgery may complain of persistent ocular discomfort or more severe ocular discomfort after successful endoscopic dacryocystorhinostomy (EDCR).

The purpose of this study was to evaluate the prevalence of DES after successful EDCR in patients with NLDO and establish screening criteria to find DES in NLDO patients before surgery.

## Methods

This study was a retrospective, observational, comparative study. The study was approved by the institutional review board of Chungnam National University Hospital (CNUH IRB 2018–11–001) and was in compliance with the tenets of the Declaration of Helsinki. The requirement for obtaining informed patient consent was waived due to the retrospective nature of the study. All data were fully anonymized before analysis and analyzed from March 2019 to December 2019.

### Patients

This study included patients who underwent EDCR for primary acquired NLDO between February 2016 and August 2018 at Chungnam National University Hospital. Patients who achieved anatomical success at 6 months after surgery with complete resolution of epiphora were included. Patients with previous history of lacrimal surgery, punctal occlusion, or canalicular obstruction were excluded in this study.

### Surgical technique

All surgeries were performed under general anesthesia. After the nasal mucosa on the lacrimal crest was incised with a Colorado monopolar cautery, the mucosa was removed using an elevator and ethmoid forceps. If necessary, uncinectomy or middle turbinectomy was performed. The lacrimal bone and maxillary bone containing the lacrimal fossa were sufficiently removed using Ferris-Smith Kerrison Rongeur forceps. The medial wall of the exposed lacrimal sac was incised with a crescent knife and removed using ethmoid forceps. After bicanalicular silicone intubation, we tied the silicone tube and inserted absorbable NasoPore® Standard (Polyganics, Groningen, Netherlands) nasal packing into the nose. The inserted silicone tube was removed 3 months after surgery.

### Examinations

OSDI questionnaires translated into Korean [7, 8] were completed by one dedicated staff member before and 4 months after EDCR. The OSDI questionnaire consisted of 12 items: 5 involved ocular symptoms, 4 involved limitations in performance, and 3 involved environmental triggers. Each item was scored on a scale of 0 to 4, depending on the frequency of symptoms experienced during the previous week. If not applicable, “Not applicable (NA)” was selected. The overall score was averaged and multiplied by 25 to convert to a 100-point scale. The OSDI is scored on a scale of 0 to 100 and higher scores represent more severe symptoms. The total OSDI score was then calculated based on the following formula:

OSDI score = (sum of scores for all items answered × 25) / (total number of items answered)

To measure TBUT, fluorescein dye was applied to the patient’s lower conjunctiva and the eyelids were opened by the examiner to prevent eye blinking. Tear film was examined using a broad blue light of slit lamp for the appearance of the first dry spots on the cornea. To perform the Schirmer’s test, a 5 × 35 mm Whatman filter paper strip was inserted into the lateral lower conjunctival sac without an anesthetic eyedrop. The length of the wetted paper was measured after 5 minutes. The DES test was performed by a single examiner in the same manner and sequence. The examiner measured TBUT, completed the OSDI questionnaire, and 5 minutes later performed the Schirmer’s test.

In this study, TFOS DEWS II criteria was used to diagnose DES. DES was defined when the OSDI questionnaire score was higher than 13 points and TBUT was less than 10 seconds [9]. Patients were divided into two groups: DES group (patients who met DES diagnosis criteria at 4 months after surgery) and control group (remaining patients).

### Statistical analyses

We analyzed the demographic and perioperative data using SPSS statistical software version 24.0 (SPSS, Chicago, IL, USA) for Microsoft Windows platform. Receiver operating characteristic (ROC) curves were computed to evaluate predictivities of screening criteria. The cut-off value, specificity and positive predictive values (PPV) were found on the ROC curve at the 75% sensitivity point. The area under the ROC curves (AUCs) was calculated according to screening criteria. We compared the AUCs using MedCalc Statistical Software for Windows version 19.2.6 (MedCalc, Ostend, Belgium). Statistical significance was set at a value of p < 0.05.

## Results

**Fig 1:**
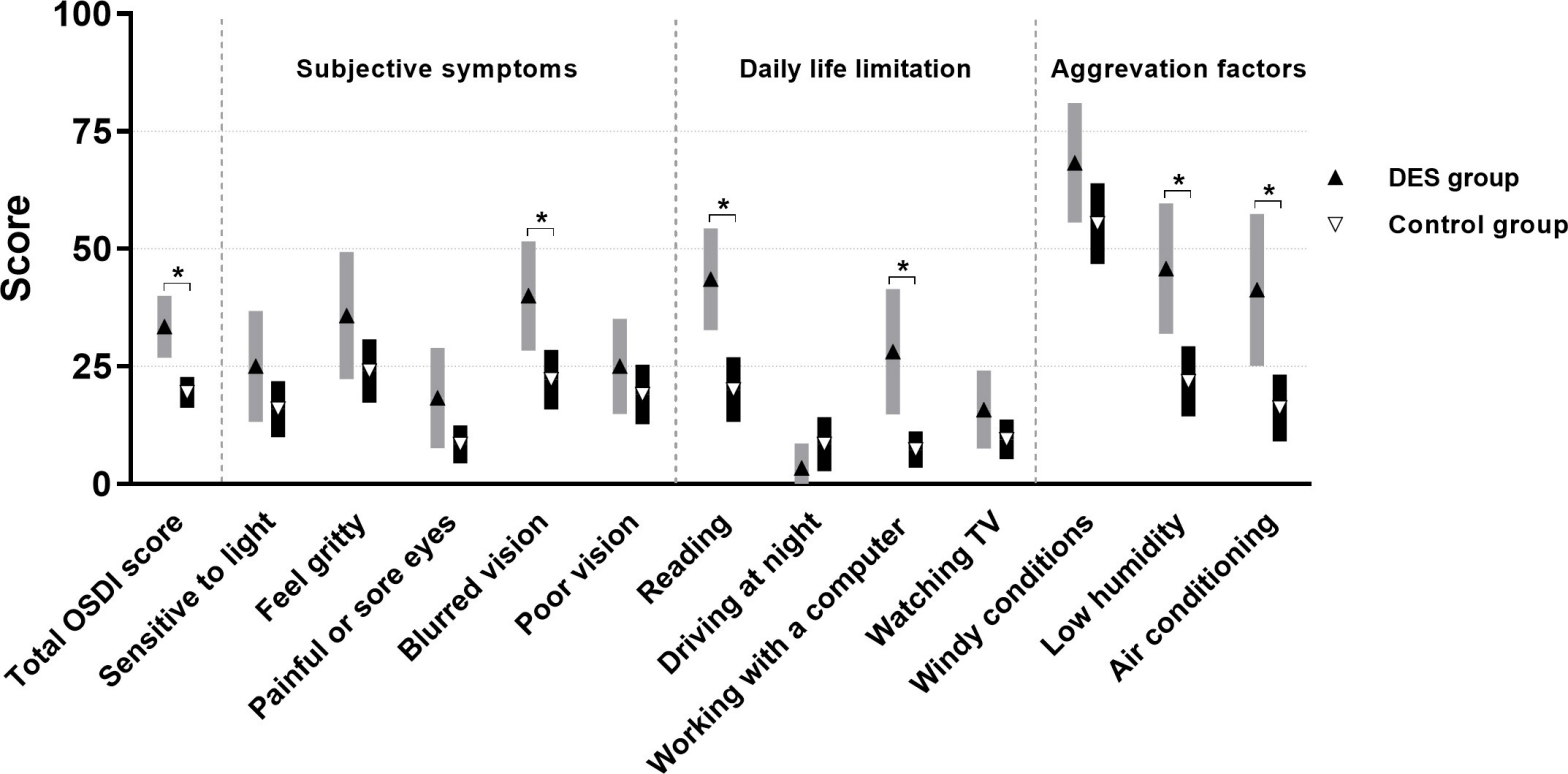
Differences in preoperative Ocular Surface Disease Index (OSDI) scores in the dry eye syndrome group and control group (each error bar denotes a 95% confidence interval of the mean). There were significant differences in the total OSDI score (p < 0.001) and the score of 5 items (blurred vision (p = 0.005), reading (p = 0.001), working with a computer (p = 0.005), low humidity (p = 0.001), and air conditioning (p = 0.006)) between the two groups before surgery. * p < 0.05, Student’s *t*-test.

A total of 110 patients were included in the study: 25 males (22.7%) and 85 females (77.3%). The mean age was 59.3 ± 13.1 years. After EDCR, 30 patients (27.3%) were diagnosed with DES (DES group), while 80 patients (72.7%) were not (control group). The incidence of DES was significantly higher in female patients (32.9% vs. 8.0%, p = 0.020). Systemic diseases that can affect dry eye conditions such as diabetes and hypertension showed no difference in both groups. Other diseases including rheumatic diseases or connective tissue diseases were not shown in our patients. The preoperative Schirmer’s and TBUT tests did not differ between the two groups, but the preoperative OSDI score was higher in DES group than in control group (34.5 vs. 19.4, p < 0.001, Table 1). When comparing each OSDI items between two groups, preoperative scores of blurred vision (p = 0.005), reading (p = 0.001), working with a computer (p = 0.005), low humidity (p = 0.001), and air conditioning (p = 0.006) were significantly higher in DES group (Fig 1). The other 7 items showed no significant difference between the two groups.

**Table 1:** Demographics and preoperative data in the DES and control groups.

Characteristics	Total	DES group	Control group	*p*-value
**No. of patients** (n, %)	110 (100)	30 (27.3)	80 (72.7)	
**Age** (years, mean ± SD)	59.3 ± 13.1	60.5 ± 13.8	58.9 ± 12.9	0.574^*^
**Sex** (n, %)				
Male	25 (22.7)	2 (6.7)	23 (28.8)	**0.020**^†^
Female	85 (77.3)	28 (93.3)	57 (71.2)	
**Diabetes** (n, %)	11 (10.0)	1 (3.3)	10 (12.5)	0.283^†^
**Hypertension** (n, %)	33 (30.0)	8 (26.7)	25 (31.3)	0.218^‡^
**TBUT** (s, mean ± SD)	7.4 ± 4.0	7.3 ± 3.9	7.4 ± 4.1	0.851^*^
**Schirmer’s test** (mm, mean ± SD)	17.5 ± 9.2	18.7 ± 9.4	17.0 ± 9.1	0.409^*^
**OSDI score** (mean ± SD)	23.3 ± 16.8	34.5 ± 17.7	19.4 ± 14.9	< 0.001^*^

DES, dry eye syndrome; SD, standard deviation; DM, diabetes mellitus; TBUT, tear film break-up time; OSDI, ocular surface disease index.

*Student’s *t*-test.

^†^Fisher’s exact test.

^‡^ Pearson Chi-Square test

Boldface values indicate statistically significant differences at p < 0.05

After surgery, the OSDI scores decreased significantly from 23.3 points to 12.3 points (p < 0.001, Table 2). The TBUT did not change (p = 0.251), but Schirmer’s test significantly decreased after EDCR (p = 0.001). There was a significant positive correlation between preoperative and postoperative OSDI score (p < 0.001); however, TBUT and Schirmer’s test did not show correlations before and after surgery (p = 0.596, p = 0.668, respectively).

**Table 2:** Changes in OSDI scores, TBUT and Schirmer’s test before and after endoscopic dacryocystorhinostomy of 110 patients.

Parameters	Preoperative (Mean ± SD)	Postoperative (Mean ± SD)	Paired *t*-test (*p-*value)	Pearson correlation (*p-*value)
**OSDI score** (mean ± SD)	23.3 ± 16.8	12.3 ± 12.7	**< 0.001**	**0.425 (< 0.001)**
**TBUT** (sec, mean ± SD)	7.4 ± 4.0	6.9 ± 2.8	0.251	0.051 (0.596)
**Schirmer’s test** (mm, mean ± SD)	17.5 ± 9.2	13.8 ± 8.6	**0.001**	0.043 (0.668)

OSDI, ocular surface disease index; TBUT, tear film break-up time

Boldface values indicate statistically significant differences at p < 0.05

We tried to establish screening criteria to predict DES that present after EDCR. Criteria A was set the same as DES diagnostic criteria (OSDI score was ≥ 13.0 and TBUT < 10 seconds). Fifty-eight patients met Criteria A preoperatively. Among them, 22 patients (37.9%) were diagnosed with DES postoperatively. Although 52 patients did not meet Criteria A, 8 patients of them (15.4%) were diagnosed with DES postoperatively. With Criteria A, DES can be screened with 73.3% sensitivity and 55.0% specificity. With the prevalence of DES assumed as 27.3% in this study, the PPV was 38.0%.

**Fig 2:**
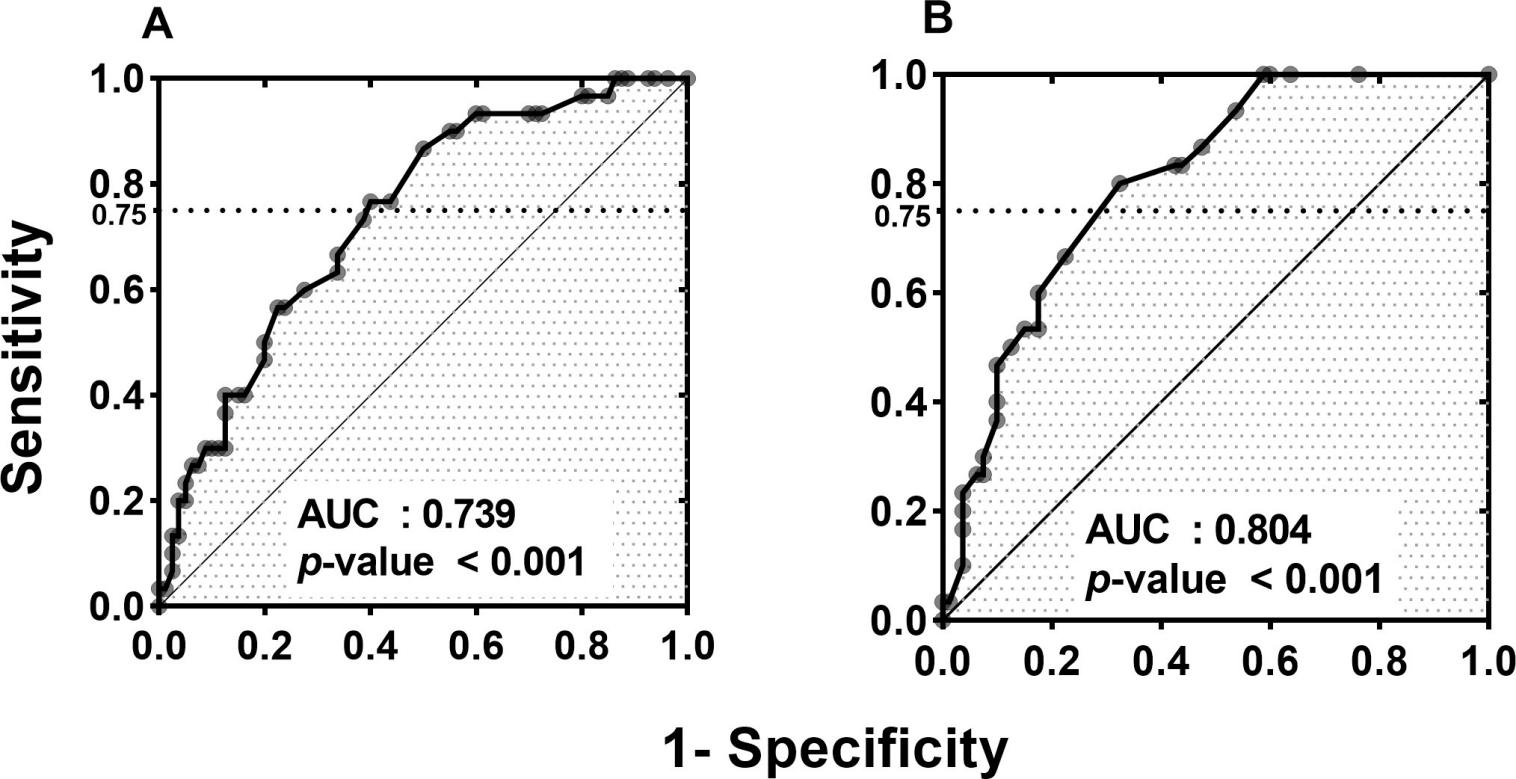
The Receiver Operating Characteristic (ROC) curve of postoperative dry eye syndrome. (A) With all 12 items of ocular surface disease index (Criteria B) and (B) with 5 selected items (blurred vision, reading, working with a computer, low humidity, or air-conditioning; Criteria C). Both Criteria B and C show significant AUC (p < 0.001, Mann-Whitney U test). AUC of Criteria C shows significantly excellent predictive ability than that of Criteria B (p = 0.0037, Pairwise comparison of ROC curves). AUC, area under the ROC curve.

Other criteria were adjusted to improve specificity and PPV while maintaining similar sensitivity to Criteria A (Fig 2). Criteria B was defined using only OSDI score without TBUT because TBUT did not show any difference in either group before and after surgery. The sensitivity was set to 75% to compare with Criteria A. Over 19.6 points of OSDI, DES was diagnosed with specificity of 60.6%, PPV of 41.7% and AUC of 0.739 (p < 0.001). Criteria C was created by selecting OSDI questionnaire items that showed significant differences before EDCR between the two groups (blurred vision, reading, working with a computer, low humidity, and air conditioning). When the sensitivity is set to 75%, the modified OSDI score is set to over 24.4 points correspondingly. At the point, Criteria C showed specificity of 71.3%, PPV of 49.5%, and AUC of 0.804 (p < 0.001). The AUC of Criteria C was significantly higher than that of Criteria B (p = 0.0037).

## Discussion

Despite successful EDCR, some patients complain of persistent discomfort or more severe ocular symptoms after surgery, some of which may be related to symptoms of DES. DES may or may not be related to EDCR, or may exist prior to surgery. Several situations may be possible for the relationship between DES and EDCR. First, EDCR induces DES; second, DES occur independently after EDCR; lastly, DES symptoms that were obscured by NLDO appear after EDCR.

If EDCR causes DES, the prevalence of DES after EDCR would be higher than that of the general population. However, the incidence of DES after EDCR in this study was 27.3%, which is not different from the prevalence of DES in the general population [2, 10–12]. If DES occurs independently after EDCR, preoperative tests should be similar in the DES group and control group. However, preoperative OSDI scores showed significant differences between the two groups, and these results suggest that DES did not occur independently after EDCR. In addition, the preoperative OSDI scores decreased significantly in both groups while maintaining correlation between preoperative and postoperative OSDI scores. This supports that the DES symptoms were obscured by NLDO and emerge after EDCR. In addition, previous studies reported that DES is one of the risk factors for NLDO [13, 14]. Therefore it is possible that NLDO occurred in some of the patients who already had had DES, and NLDO improved after EDCR, but DES remained.

In this study, the patients diagnosed with DES have short TBUT and normal range of the Schirmer’s test, which correspond with evaporative DES [1]. Evaporative DES is caused by inflammation of the eyelids and ocular surface and meibomian gland dysfunction is the most common cause [1]. The etiology of primary acquired NLDO is not clear, but some studies suggested that it is caused by chronic inflammation of the lacrimal sac and nasolacrimal duct [13]. The fact that inflammation is commonly associated with NLDO and meibomian gland dysfunction suggests that both disease can occur together. However, thickened tear meniscus due to NLDO can offset the symptoms of evaporative DES, making it difficult to diagnose DES before EDCR.

The authors tried to find standard criteria for screening the possibility of DES hidden by NLDO preoperatively. In this study, the combination of OSDI score and TBUT was used as the diagnostic criteria for DES according to the TFOS DEWS II report [9]. TBUT is a test that evaluates tear film layer instability, known to have high sensitivity but low specificity [15, 16]. In this study, 73 patients had an OSDI score less than 13 points postoperatively, and 56 of them (76.7%) had a TBUT of less than 10 seconds. In other words, tear film instability could exist even without DES symptoms, relating to the low specificity of the TBUT. Additionally, there was no significant difference in TBUT before and after surgery in both the DES group and the control group and this indicates that TBUT is a test with low diagnostic value. The OSDI questionnaire is a test that measures subjective symptoms and is widely used in DES diagnosis. This test is simple yet organized with an excellent ability to discriminate DES [9]. In the present study, the preoperative OSDI score was significantly higher in the DES group than in the control group, and it was found to decrease significantly after surgery. Therefore, OSDI questionnaire was more appropriate than TBUT for DES screening before EDCR.

Criteria A was set the same as DES diagnostic criteria, and it showed 73.3% sensitivity, 55.0% specificity, and 38.0% PPV. Criteria A showed relatively high sensitivity, but the specificity and the positive predictive rate were insufficient. As mentioned above, OSDI questionnaire was more useful than TBUT to screen DES before EDCR. We drew the ROC curve and set Criteria B using only OSDI questionnaire. The cut-off value was adjusted so that the sensitivity was set to 75%. At the 19.6 points of cut-off value, the Criteria B showed 75% sensitivity, 60.6% specificity, and 41.7% PPV (AUC = 0.739, p < 0.001). By subtracting TBUT from Criteria A, specificity and PPV were improved.

We composed criteria by selecting some of the OSDI items in order to find screening criteria showing higher specificity and PPV. The OSDI questionnaire consists of 12 items covering ocular discomfort, daily life limitation, and aggregation factors. Preoperative OSDI scores’ distributions were different in the DES and control groups, indicating a significant difference in blurred vision (p = 0.005), reading (p = 0.001), working with a computer (p = 0.005), low humidity (p = 0.001), and air conditioning (p = 0.006). These OSDI questionnaire items resulted in improving the AUC value. We drew the ROC curve and set Criteria C using these five OSDI questionnaire items. At 75% sensitivity, the cut-off value was 24.4 and Criteria C showed 71.3% specificity and 49.5% PPV (AUC = 0.804, p < 0.001). Although both Criteria B and Criteria C showed AUCs over 0.7, the AUC of Criteria C was significantly higher than that of Criteria B (p = 0.0037).

This is the first study to predict DES occurrence in patients with NLDO before EDCR. However, there were some limitations. First, because DES symptoms change over time, the diagnosis should be made by considering long-term variations rather than a single test. Secondly, the prevalence of DES varies according to sex and age, but these parameters were uncontrolled in this study. In addition, corneal epitheliopathy, an important pathological mechanism of DES, was not included in the diagnostic criteria of DES in the study. However, corneal epitheliopathy was seen only in a small number of patients in this study because of thick tear meniscus in patients with NLDO, making it difficult and inappropriate to include corneal staining in the diagnostic criteria of DES. Recently, Asia Dry Eye Society [17] reported that it is more useful to diagnose DES with only reduced TBUT and DES symptoms through OSDI or DEQ-5. Taking these two into account, corneal epitheliopathy may not be essential when diagnosing DES in NLDO patients. Lastly, non-invasive TBUT is more commonly recommended for DES diagnosis [9], but in this study TBUT using fluorescein dye was performed because screening tests was done in an oculoplastic clinics. Additional research on screening criteria using non-invasive TBUT would be needed.

In conclusion, DES appeared in 27.3% of the patients after successful EDCR, implying that the concurrent presence of NLDO and DES is not an unusual condition. The OSDI questionnaire can be suitably used to predict DES that appears after surgery, and more accurate prediction is possible by using modified OSDI, which selectively used five items of OSDI.

## Supporting information

S1 Table Data for all subjects included in this study showing clinical results. (XLSX)

## References

[1] Craig JP, Nichols KK, Akpek EK, Caffery B, Dua HS, Joo CK, et al. TFOS DEWS II Definition and Classification Report. Ocul Surf. 2017;15(3):276–83. Epub 2017/07/25. 10.1016/j.jtos.2017.05.008 . 28736335

[2] Lee A, Lee J, Saw S, Gazzard G, Koh D, Widjaja D, et al. Prevalence and risk factors associated with dry eye symptoms: a population based study in Indonesia. Br J Ophthalmol. 2002;86(12):1347–51. 10.1136/bjo.86.12.1347 12446361 PMC1771386

[3] Lee-Wing MW, Ashenhurst ME. Clinicopathologic analysis of 166 patients with primary acquired nasolacrimal duct obstruction. Ophthalmology. 2001;108(11):2038–40. 10.1016/s0161-6420(01)00783-7 11713075

[4] Stahl U, Francis IC, Stapleton F. Prospective controlled study of vapor pressure tear osmolality and tear meniscus height in nasolacrimal duct obstruction. Am J Ophthalmol. 2006;141(6):1051–6. 10.1016/j.ajo.2005.12.051 16765672

[5] Lee J, Kim T. Changes in cytokines in tears after endoscopic endonasal dacryocystorhinostomy for primary acquired nasolacrimal duct obstruction. Eye. 2014;28(5):600. 10.1038/eye.2014.33 24577250 PMC4017116

[6] Yuksel N, Akcay E, Ayan B, Duru N. Tear-film osmolarity changes following dacryocystorhinostomy in primary acquired nasolacrimal duct obstruction. Curr Eye Res. 2017;42(3):348–50. 10.1080/02713683.2016.1196706 27419611

[7] Walt J, Rowe M, Stern K. Evaluating the functional impact of dry eye: the Ocular Surface Disease Index. Drug Inf J. 1997;31(1436):b5.

[8] Her J, Yu S-I, Seo S-G. Clinical effects of various antiinflammatory therapies in dry eye syndrome. Journal of the Korean Ophthalmological Society. 2006;47(12):1901–10.

[9] Wolffsohn JS, Arita R, Chalmers R, Djalilian A, Dogru M, Dumbleton K, et al. TFOS DEWS II Diagnostic Methodology report. Ocul Surf. 2017;15(3):539–74. Epub 2017/07/25. 10.1016/j.jtos.2017.05.001 . 28736342

[10] Doughty MJ, Fonn D, Caffery B, Gordon K, Richter D, Simpson T. the Canadian Dry Eye Epidemiology Study (candees), A First Report: poster# 92 (cl-307). Optom Vis Sci. 1995;72(12):154.

[11] Moss SE, Klein R, Klein BE. Prevalence of and risk factors for dry eye syndrome. Arch Ophthalmol. 2000;118(9):1264–8. 10.1001/archopht.118.9.1264 10980773

[12] Schein OD, MUÑO B, Tielsch JM, Bandeen-Roche K, West S. Prevalence of dry eye among the elderly. Am J Ophthalmol. 1997;124(6):723–8. 10.1016/s0002-9394(14)71688-5 9402817

[13] Nemet AY, Vinker S. Associated morbidity of nasolacrimal duct obstruction—a large community based case–control study. Graefe’s Archive for Clinical and Experimental Ophthalmology. 2014;252(1):125–30. 10.1007/s00417-013-2484-3 24146269

[14] Woog JJ. The incidence of symptomatic acquired lacrimal outflow obstruction among residents of Olmsted County, Minnesota, 1976–2000 (an American Ophthalmological Society thesis). Trans Am Ophthalmol Soc. 2007;105:649–66. 18427633 PMC2258133

[15] Lemp MA, Bron AJ, Baudouin C, del Castillo JMB, Geffen D, Tauber J, et al. Tear osmolarity in the diagnosis and management of dry eye disease. Am J Ophthalmol. 2011;151(5):792–8. e1. 10.1016/j.ajo.2010.10.032 21310379

[16] Vitali C, Moutsopoulos HM, Bombardieri S. The European Community Study Group on diagnostic criteria for Sjögren’s syndrome. Sensitivity and specificity of tests for ocular and oral involvement in Sjögren’s syndrome. Ann Rheum Dis. 1994;53(10):637. 10.1136/ard.53.10.637 7979575 PMC1005429

[17] Tsubota K, Yokoi N, Shimazaki J, Watanabe H, Dogru M, Yamada M, et al. New perspectives on dry eye definition and diagnosis: a consensus report by the Asia Dry Eye Society. The ocular surface. 2017;15(1):65–76. 10.1016/j.jtos.2016.09.003 27725302

